# Astrocyte Dysfunction Reflected in Ischemia-Induced Astrocyte-Derived Extracellular Vesicles: A Pilot Study on Acute Ischemic Stroke Patients

**DOI:** 10.3390/ijms252212471

**Published:** 2024-11-20

**Authors:** Timea Forró, Doina Ramona Manu, Lucian Barbu-Tudoran, Rodica Bălașa

**Affiliations:** 1 Doctoral School of Medicine and Pharmacy, “George Emil Palade” University of Medicine, Pharmacy, Science and Technology of Targu Mures, 540142 Targu Mures, Romania; forro.btimea@gmail.com; 2 Center for Advanced Medical and Pharmaceutical Research, “George Emil Palade” University of Medicine, Pharmacy, Science and Technology of Targu Mures, 540142 Targu Mures, Romania; 3 Electron Microscopy Laboratory, Department of Molecular Biology and Biotechnology, Faculty of Biology and Geology, Babes-Bolyai University, 400006 Cluj-Napoca, Romania; lucian.barbu@ubbcluj.ro; 4 Electron Microscopy Integrated Laboratory, National Institute for Research and Development of Isotopic and Molecular Technologies, 400293 Cluj-Napoca, Romania; 5 Department of Neurology, “George Emil Palade” University of Medicine, Pharmacy, Science and Technology of Targu Mures, 540142 Targu Mures, Romania; rodica.balasa@umfst.ro; 6 1st Neurology Clinic, County Emergency Clinical Hospital of Targu Mures, 540136 Targu Mures, Romania

**Keywords:** acute ischemic stroke, astrocyte-derived extracellular vesicles, aquaporin-4, glial cell line-derived neurotrophic factor, Western blotting

## Abstract

Extracellular vesicles (EVs) secreted by astrocytes (ADEVs) mediate numerous biological processes, providing insights into damage, repair, and protection following ischemic stroke (IS). This pilot study aimed to broaden the current knowledge on the astrocyte response to ischemia by dynamically assessing the aquaporin-4 (AQP4) and glial cell line-derived neurotrophic factor (GDNF) as cargo proteins of these vesicles in eighteen acute IS patients and nine controls. EV proteins were detected by Western blotting and followed 24 h (D1), 7 days (D7), and one month (M1) after symptoms onset. The post-ischemic ADEV AQP4 and GDNF levels were higher at D1 compared to the control group (*p* = 0.006 and *p* = 0.023). Significant differences were observed in ADEV AQP4 during the three evaluated time points (n = 12, *p* = 0.013) and between D1 and D7 (z = 2.858, *p* = 0.012), but not in EV GDNF. There was a positive relationship between the severity of stroke at D1 according to the National Institutes of Health Stroke Scale, and ADEV AQP4 at D1 (r = 0.50, *p* = 0.031), as well as ADEV GDNF at D1 and D7 (r = 0.49, *p* = 0.035 and r = 0.53, *p* = 0.021, respectively). The release of EVs with distinct protein profiles can be an attractive platform for the development of biomarkers in IS.

## 1. Introduction

Ischemic stroke (IS) is a leading cause of mortality and long-term disability worldwide [1,2]. Despite advancements in acute-phase treatment with intravenous thrombolysis (IVT) and mechanical thrombectomy, up to ≥50% of stroke patients treated within the time window do not have favorable outcomes [3]. Commonly used neuroimaging techniques, such as magnetic resonance imaging and computed tomography (CT), have notably improved our ability to visualize brain structures [4], but they do not provide insights into the underlying disease’s biology or the status of microcirculation.

Astrocytes are the most abundant glial cell type in the central nervous system (CNS), with remarkable heterogeneity in structure and function. Under physiological conditions, they play a critical role in maintaining brain metabolic, ion-water, and neurochemical homeostasis, but they also support essential neuronal functions, regulate blood–brain barrier (BBB) permeability, and facilitate synapse formation and plasticity [5,6,7,8]. In response to brain damage, astrocytes undergo various biochemical and spatiotemporal changes that lead to hypertrophy of their main cellular processes and alterations in their protein profiles [9]. Reactive astrocytes (RAs) are more resistant to ischemia than neurons [10,11]. Therefore, they hold the potential to facilitate functional recovery, restoring neuronal integrity, particularly in the ischemic penumbra [12].

Recent evidence indicates that astrocytes may gain their functions through the release of extracellular vesicles (EVs) [13]. EVs are lipid-bilayer membrane-enclosed particles that are secreted by all types of cells. They can be detected in all tissues and bodily fluids and can freely pass the BBB. EVs transport various biological components (e.g., proteins, lipids, and nucleic acids) between cells as a form of intercellular communication, influencing numerous physiological and pathological processes, including those occurring under ischemic conditions [14,15,16]. Ischemic-preconditioned astrocytes act like a “double-edged sword” in IS and secrete EVs with both neuroprotective and neurotoxic effects on the CNS [13,17]. Additionally, they respond to different extracellular stimuli and changes in the microenvironment by modifying the cargo of these EVs [18,19,20]. Furthermore, these vesicles carry disease-specific molecular signatures that reflect their cells of origin, making them an ideal source of non-invasive biomarkers of stroke progression and recovery, which holds considerable potential for various clinical applications [14,21].

Aquaporin-4 (AQP4) is the main water channel of the CNS, predominantly located on perivascular astrocyte endfeet, crucial for maintaining brain water homeostasis [21,22,23]. After an ischemic event, it becomes upregulated in the brain [24,25] and is associated with the timing of both the formation and resolution of cerebral edema [21]. Additionally, it has a protective role on stroke outcomes, as previously suggested by various preclinical studies investigating its impact on the ischemic brain using AQP4 knockout mice [26,27,28]. However, controversies still exist; AQP4 deficiency has been reported to reduce neuroinflammation and improve neuronal survival without directly affecting edema formation [29,30]. In a clinical context, AQP4 has been detected in the circulation of tissue Plasminogen Activator (t-PA)-treated IS patients, highlighting its potential as a biomarker for neurological recovery in the acute–subacute phase post-symptoms onset [31].

Discovered in the early 1990s, the glial cell line-derived neurotrophic factor (GDNF) is a potent growth, survival, and neurotrophic factor [32]. It is mainly expressed by neurons in a healthy brain, but it is upregulated and released by glial cells in response to ischemia [33]. Experimental transient focal and global ischemia models also revealed increased levels of GDNF [34]. Furthermore, there is evidence suggesting that GDNF derived from RAs promotes neuronal survival and facilitates brain repair and recovery, ultimately leading to improved long-term outcomes following cerebral ischemia [35]. It also alleviates ischemia-induced learning and memory disorders after a stroke [36].

While studies profiling the cargo of different post-ischemic brain-derived EVs are continuously emerging, only a minority focus on their protein content and link it to specific cell types within the CNS [37]. In a previous pilot study, we explored the glial fibrillary acidic protein (GFAP) as cargo protein of astrocyte-derived extracellular vesicles (ADEVs) in patients with acute ischemic stroke (AIS) at 24 h (D1), 7 days (D7), and one month (M1) following symptoms onset [38]. Here, we aimed to extend our research concerning the post-ischemic behavior of astrocytes by detecting and characterizing two more cargo proteins, AQP4 and GDNF, in these EVs within the same patient group. As far as we are concerned, this is one of the first studies quantifying the AQP4 and GDNF within ADEV cargo of IS patients. We also aimed to assess the biomarker potential of these EV proteins in determining stroke severity and outcome.

## 2. Results

### 2.1. Study Population

The study population’s clinical characteristics were previously presented in our recent article [38]. Briefly, we enrolled eighteen AIS patients within 24 h of symptom onset, with a mean age at the study inclusion of 66 ± 7.5 years (ranging from 51 to 78). A total of 50% of stroke participants received IVT. The median clinical stroke severity on the National Institutes of Health Stroke Scale (NIHSS) was 7.5 (ranging from 6 to 11) at D1, 5.5 (ranging from 1 to 10) at D7, and 2 (ranging from 1 to 7) at M1. The TOAST classifications were as follows: 77.7% large artery atherosclerosis and 22.3% cardioembolic. According to the modified Rankin Scale (mRS), over 80% of the patients presented moderate to severe disability at D7 that improved by M1. In the control group, we included nine subjects with a mean age of 65 ± 7 years (ranging from 53 to 74), with similar demographic and cardiovascular risk factor profiles to the target group. Table 1 summarizes the baseline characteristics of the study’s population. During follow-up, six patients missed their one-month blood samples as they could not manage to present for various reasons, such as transfer to other neurorehabilitation facilities, reinfarction, and SARS-CoV-2 infection.

### 2.2. Western Blot Analyses of EV Cargo Proteins

We performed Western blotting on the protein lysates from the total extracellular vesicle (TEV) and ADEV aliquots to detect and quantify the AQP4 and GDNF in EV cargo from the stroke-related samples compared to the control group.

**Figure 1:**
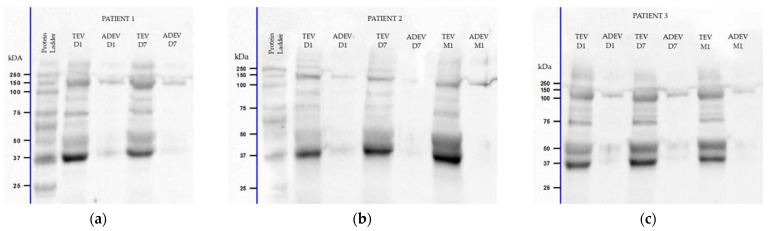
Representative Western blots for aquaporin-4 (AQP4) showing multiple bands in extracellular vesicle (EV) aliquots of an AIS patient with two key moments that missed one-month follow-up (**a**) and two patients with three key moments (**b**,**c**): one major band at ≈37 kilodaltons (kDa), additional bands at 50 and 75 kDa, suggesting AQP4 dimers or glycosylation, and between 100–150 kDa as possible tetramers. Approximate molecular weight (MW) markers in kDa are labeled adjacent on the left. Abbreviations: TEV—total extracellular vesicles; ADEV—astrocyte-derived extracellular vesicles; D1—24 h; D7—7 days; M1—one month after stroke onset.

When the blots were probed with the anti-AQP4 antibody, a prominent band in TEVs and a discrete one in ADEVs at around 37 kilodaltons (kDa) were detected in all post-stroke samples obtained at D1, D7, and M1 after injury. These bands may correspond to the AQP4 protein, which has a reported molecular weight (MW) of ≈35–45 kDa. An additional band between the 100 and 150 kDa MWs was also visible, suggesting a possible upregulation of the AQP4 tetramers in EV cargo. Additionally, two other bands were observed near 50 and 75 kDa in a few patients, suggesting the potential presence of AQP4 dimers or glycosylation (Figure 1).

**Figure 2:**
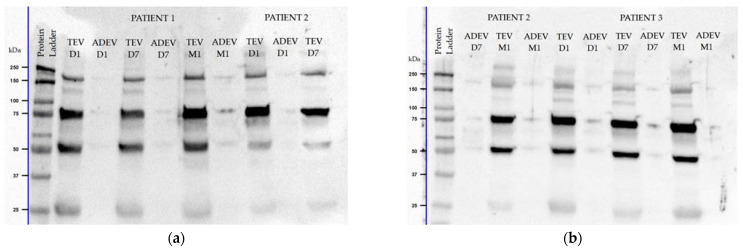
Representative Western blots for glial cell line-derived neurotrophic factor (GDNF) of three AIS patients with three key moments (**a**,**b**). Data for patient 2 is presented on separate blots: D1 and D7 TEV (**a**), D7 ADEV and M1 (**b**). The blots display multiple bands in EV aliquots: at ≈25 kDa as a monomer, at ≈50 kDa as a dimer, and, additionally, near 75 and 150 kDa MW as a combination of a monomer and dimer**.** Approximate MW markers in kDa are labeled adjacent on the left.

When the blots were probed with the anti-GDNF antibody, two major bands were detected in the post-ischemic EVs. One band appeared around 50 kDa, likely representing a dimer or glycosylated form of GDNF, while another one, at 75 kDa, was possibly a mixture of GDNF monomer, dimer, and GDNF receptor-α [39]. Furthermore, additional bands were observed at 25 kDa, corresponding to the predicted MW of GDNF (mainly detected in TEVs), and at 150 kDa, which may represent dimers of the 70–85 kDa complex [39] (Figure 2).

### 2.3. EV AQP4 and EV GDNF in AIS Patients and Controls

To assess the impact of brain ischemia on the expression of AQP4 and GDNF in EV cargo, we analyzed these proteins’ band intensities in the TEVs and ADEVs of AIS patients (D1, D7: n = 18, M1: n = 12) and compared them to those of the control subjects (n = 9). For the analyses, we focused on the ≈37 kDa AQP4 and ≈50 kDa GDNF dimer.

**Figure 3:**
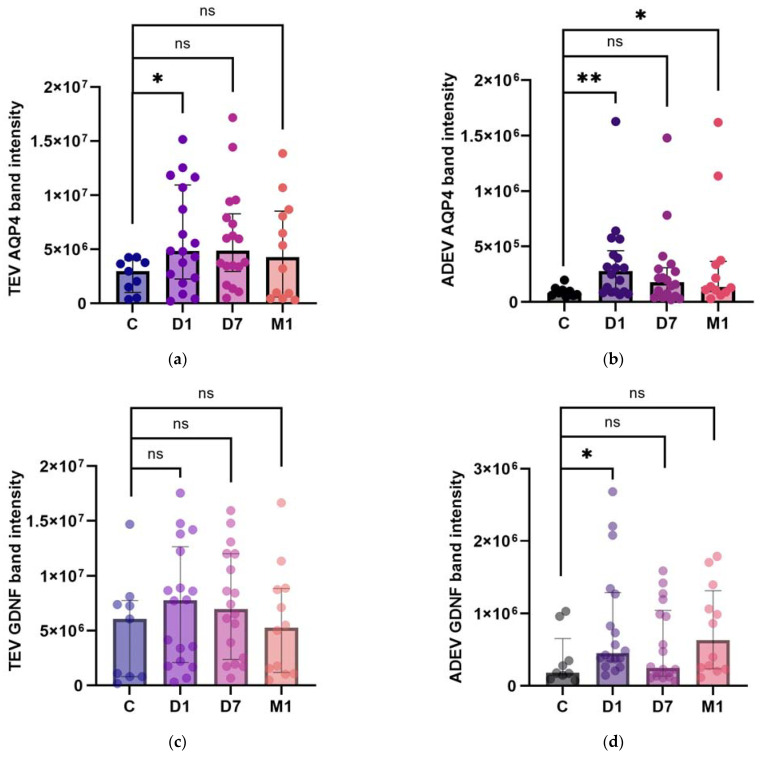
AQP4 (**a**,**b**) and GDNF (**c**,**d**) band intensities in TEVs (**a**,**c**) and ADEVs (**b**,**d**) of AIS patients and control participants (C). Data are represented as individual value boxplots with median and interquartile range (IQR, Mann–Whitney U test; * *p*  <  0.05; ** *p*  <  0.01; ns—not significant).

Densitometric analysis of the immunoblots indicated that the levels of AQP4 were significantly higher in stroke patients at D1 compared to the controls, both in the TEVs [median × 10^6^ (interquartile range, IQR × 10^6^): 4.83 (2.46–10.21) vs. 2.96 (1.51–3.76), *p* = 0.047] and ADEVs [median × 10^6^ (IQR × 10^6^): 0.27 (0.09–0.41) vs. 0.08 (0.06–0.1), *p* = 0.006]. During the follow-up, we observed a significant increase in patients’ AQP4 levels, but only in the ADEVs at M1 [median × 10^6^ (IQR × 10^6^), TEVs at D7 and M1, respectively: 4.89 (3.4–7.78) vs. 2.96 (1.51–3.76), *p* = 0.067; 4.29 (0.82–8.23) vs. 2.96 (1.51–3.76), *p* = 0.463; ADEVs at D7 and M1, respectively: 0.17 (0.05–0.29) vs. 0.08 (0.06–0.1), *p* = 0.180; 0.13 (0.1–0.34) vs. 0.08 (0.06–0.1), *p* = 0.049] (Figure 3a,b).

The increase in the GDNF did not reach the level of significance in the TEVs of stroke patients at D1, D7, and M1 compared to the controls [median × 10^6^ (IQR × 10^6^), D1: 7.78 (2.5–11.41) vs. 6.07 (0.8–7.39), *p*  =  0.212; D7: 6.97 (2.87–11.64) vs. 6.07 (0.8–7.39), *p*  =  0.173; M1: 5.27 (1.43–8.79) vs. 6.07 (0.8–7.39), *p*  =  0.651]. However, we found the GDNF levels to be significantly elevated at D1 in the ADEVs [median × 10^6^ (IQR × 10^6^), D1: 0.45 (0.37–1.16) vs. 0.18 (0.14–0.34), *p* = 0.023); D7: 0.24 (0.14–0.98) vs. 0.18 (0.14–0.34), *p* = 0.375; M1: 0.63 (0.24–1.14) vs. 0.18 (0.14–0.34), *p* = 0.058] (Figure 3c,d).

Then, we divided our patients based on IVT treatment into those who received t-PA (D1, D7: n = 9, M1: n = 6) and those who did not (D1, D7: n = 9, M1: n = 6). There were no differences regarding EV AQP4 (TEVs at D1, D7, and M1, respectively: *p* > 0.999, *p* = 0.863, *p* > 0.999; ADEVs at D1, D7, and M1, respectively: *p* = 0.730, *p* = 0.604, *p* = 0.937), and EV GDNF (TEVs at D1, D7, and M1, respectively: *p* = 0.730, *p* = 0.666, *p* = 0.699; ADEVs at D1, D7, and M1, respectively: *p* = 0.222, *p* = 0.161, *p* = 0.309) between the two groups.

### 2.4. Temporal Profile of EV AQP4 and EV GDNF

**Figure 4:**
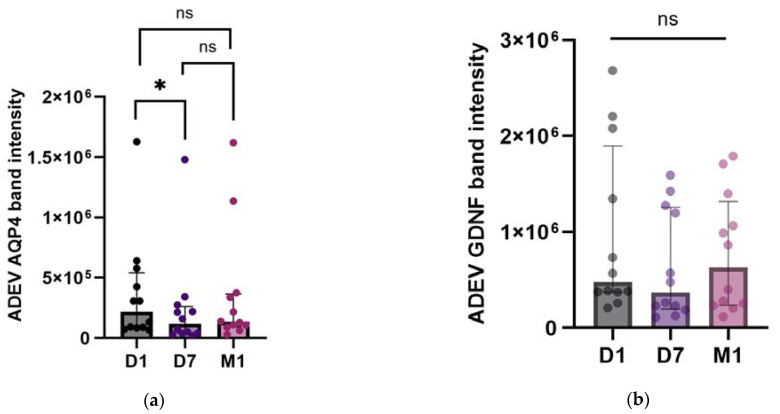
AQP4 (**a**) and GDNF (**b**) band intensities in ADEVs during the patients’ follow-up: D1, D7, and M1. Data are represented as individual value boxplots with median and IQR (Friedman’s ANOVA, followed by Dunn’s post hoc only for AQP4 (**a**), as for GDNF, there were no statistically significant differences (**b**); * *p*  <  0.05; ns—not significant).

Next, to determine whether there was a variance in the EV cargo over the evaluated time points, we compared the band intensities of AQP4 and GDNF between D1, D7, and M1 after the onset of the symptoms. Analysis by Friedman’s ANOVA test revealed a statistically significant difference in AQP4 in the ADEVs (n = 12, *p* = 0.013) but not in the TEVs (n = 12, *p* = 0.716) within the follow-up period. Further, the post hoc Dunn’s analysis showed a difference between D1 and D7 (z = 2.858, *p* = 0.012) but not between D1 and M1 or D7 and M1 in the ADEVs (Figure 4a). Regarding EV GDNF, there were no differences between the three evaluated time points (n = 12, TEVs: *p* = 0.920, ADEVs: *p* = 0.075; Figure 4b).

### 2.5. Correlations Between EV Cargo, Stroke Severity, and Functional Outcome

We subsequently explored whether the amount of AQP4 and GDNF in the EV cargo correlated with the severity of neurological deficit, assessed by the NIHSS score at D1, D7, and M1, or with the short-term outcome according to the mRS score at D7 and M1 following stroke onset. A positive relationship was observed between the NIHSS at D1 and ADEV AQP4 at D1 (r = 0.50, *p* = 0.031), as well as ADEV GDNF at D1 (r = 0.49, *p* = 0.035) and D7 (r = 0.53, *p* = 0.021). None of the evaluated parameters at any time point correlated with the mRS at D7 or M1 (Table 2).

## 3. Discussion

Astrocytes are special glial cells within the CNS and are actively involved in post-stroke neuroprotection and neurorepair by releasing EVs [40]. ADEVs preferentially increase over the first month following an IS, possibly due to their trophic support on damaged neurons [41]. This process may be an important response to brain injury whereby various extracellular stimuli can alter ADEVs‘ downstream functions and cargo to contain neuroprotective factors, stimulating neurite outgrowth and promoting neuronal survival [19,20]. While research regarding the role of ADEVs in ischemic brain injury is continuously emerging, a deeper understanding of the astrocytes and how their EV profile changes under these conditions is still needed [37]. Here, we demonstrated the presence of AQP4 and GDNF in ADEV cargo during the first month following stroke, expanding on our previous findings regarding the post-ischemic ADEV profile and the GFAP content of these vesicles [38].

### 3.1. Western Blot Profile of AQP4 and GDNF in Post-Ischemic TEVs and ADEVs

The AQP4 has an expected MW of 35–45 kDa, depending on the level of glycosylation [42]. Immunoblotting detected a prominent AQP4 band around 37 kDa MW in all aliquots of the TEVs and a discrete one in the ADEVs over the first month following injury. According to the literature, the AQP4 is expressed as two main isoforms, namely M1-AQP4 (≈34 kDa, AQP4a) and M23-AQP4 (≈31 kDa, AQP4c), generated by alternative splicing [43,44]. AQP4 monomers are organized into more complex structures, forming tetramers, which further aggregate into orthogonal arrays of particles (OAPs). These supramolecular assemblies represent building blocks of ≈240 kDa that can further form higher-order OAPs, reaching ≈1000 kDa [45]. We also detected bands between 100 and 150 kDa MW, suggesting the possible presence of AQP4 tetramers in EV cargo during stroke. It was related that the M1-AQP4 mainly exists as individual tetramers and does not aggregate into OAPs by itself. However, it can co-assemble with M23-AQP4 to form arrays as hetero-tetramers. The M23-AQP4 forms OAPs whose size depends on the ratio between M1/M23: higher levels of M1-AQP4 composition result in a reduced OAP size [46,47,48]. Changes in the M1/M23 ratio and the disintegration of OAPs were observed early after stroke, but their pathophysiological consequences are still unclear [47]. In addition, further bands were visible near the 50 and 75 kDa MWs in a few patients. Lu et al. proposed that the ≈60 kDa bands may be dimers of AQP4 [44], while Khan et al. suggested that high MW AQP4 bands ≈75 kDa may reflect glycosylation, subunit dimerization, or both [42]. Other papers on animal models reported additional AQP4 bands of 49.5 kDa in dogfish and 52 kDa in rats, both discrete bands [49,50], similar to ours near the 50 and 75 kDa MWs.

GDNF is first synthesized as a 211 amino acid precursor protein (pro-GDNF), which undergoes a series of protein cleavages and processing to be converted into the mature form [51]. This biologically active form is a glycosylated, disulfide-bonded homodimer of about 33–45 kDa MW [52,53]. As previously reported, there is a potential to observe multiple bands on the Western blots for GDNF due to the existence of five different isoforms: a signal peptide, a propeptide, as well as post-translational modifications, such as glycosylation [54,55]. We detected two major bands in EVs when the blots were probed with the anti-GDNF antibody: one band near the 50 kDa MW—likely representing a dimer or glycosylated form of GDNF—and another one at the MW of 75 kDa. Based on a previous report, bands with a high MW (70–85 kDa) may be interpreted as a mixture of the GDNF monomer and dimer cross-linked to the glycosylated GDNF receptor-α or another unknown GDNF-binding protein [39]. Additional bands were observed at 25 kDa, corresponding to the predicted MW of GDNF, and near 150 kDa, possible dimers of the 70–85 kDa complex [39]. Similarly, previous studies reported multiple bands on the GDNF blots: three major bands in head and neck squamous cell carcinoma cell lines (24 kDa as the predicted MW, 35 kDa as a probable glycosylated form, and a band right above 10 kDa as a signal peptide of GDNF) [55], two bands in human limbal epithelia (21 kDa as a mature form and 35 kDa as a glycosylated homodimer of GDNF) [54], as well as two bands in lysates from adult and neonatal mouse brains (≈36 kDa as fully glycosylated mature homodimers and ≈50 kDa as a proform homodimer of GDNF) [56].

### 3.2. Temporal Profile of EV AQP4 in AIS Patients

The involvement of AQP4 in various pathological conditions is primarily based on in vitro studies, findings from post-mortem brain tissue, and the use of AQP4-deficient rodent models [57]. In cultured cortical astrocytes, the expression of AQP4 was found to be time-dependent, probably in response to the extent of post-ischemic astrocytic damage. Following oxygen–glucose deprivation, the AQP4 concentrations immediately decrease but then increase between the first and third day, returning to normal levels after 7 days of reoxygenation [58]. In a rat pup stroke model, the induction of AQP4 in the border regions limited the edema formation that occurs later after injury (24–72 h), thus preserving the tissue. RAs showed intense AQP4 expression 72 h post-stroke, indicating the initiation of astrogliosis. AQP4 labeling in RAs continued to increase even after 28 days [59]. Pathohistological studies on post-mortem human samples revealed an upregulation of AQP4 in the glial scar during the chronic stages after stroke [24,25,60,61].

Our study indicated an ischemia-induced upregulation of EV AQP4 at 24 h and one month, but only in ADEVs, compared to the controls. Previously, Ramiro et al. observed that the serum levels of AQP4 peak within the very early phase (the first 1 to 2 h) of IVT-treated IS, but these levels were not maintained at 12 and 24 h after symptoms onset. Furthermore, the baseline circulating AQP4 levels were similar between patients and healthy controls but higher in those patients who presented early neurological improvement (defined as a ≥4-point decrease in the NIHSS score compared to the baseline score), either within the first 48 h after stroke onset or at the time of hospital discharge during the recovery phase of the disease [31]. Another study revealed that early neurological deterioration (defined as a ≥4-point difference in the 24 h post-IVT NIHSS score compared to the initial score) can be influenced by systolic blood pressure, possibly through oxidative stress-induced BBB disruption and AQP4 upregulation at 24 h after thrombolysis [62]. These studies suggest that the changes previously described in the ischemic brain can be reflected in the circulation of AIS patients.

Here, the ADEV AQP4 levels demonstrated time-dependent fluctuations over the patient’s follow-up but with no apparent impact of IVT treatment. In addition, at 24 h, these levels showed a significant correlation with the stroke’s severity. In the previously mentioned study, Ramiro et al. observed a negative correlation between the AQP4 baseline levels and the NIHSS score upon admission and the ischemic lesion growth 1–3 days after symptoms onset. Thereby, higher baseline AQP4 levels might be protective against a stroke, indicating that this protein could independently predict good neurological outcomes and be used as a biomarker of neurological recovery in the acute-subacute phase of a stroke [31]. In contrast, He et al. revealed a positive correlation between the serum AQP4 levels at 24 h post-IVT and the severity of neurological deterioration [62]. Regarding the potential link between AQP4 and stroke outcomes, we found no correlation between EV AQP4 and mRS at 7 days or one month. Ramiro et al. also assessed this aspect, but in the third month, they did not find any association between the AQP4 levels at baseline and long-term outcomes measured by the mRS [31].

### 3.3. Temporal Profile of EV GDNF in AIS Patients

Earlier studies have shown an increase in GDNF expression in the experimental models of transient focal and global ischemia. The levels of GDNF peak 2–6 h after ischemia-reperfusion, followed by a second increase at 72 h, resulting in a characteristic biphasic pattern of GDNF upregulation [63,64,65]. The early increase occurs in the cortical neurons, suggesting a temporary production of GDNF that protects the brain against injury. The secondary increase during the late post-ischemic phase may be attributed to the activation of different types of glial cells, particularly ischemia-induced RAs [63,64]. It was also revealed that both surviving neurons and ischemia-induced astrocytes produce GDNF between the third and seventh day following the onset of ischemia [64].

We observed higher levels of GDNF at 24 h in post-stroke ADEVs compared to the controls, with no differences between the three evaluated time points and no apparent impact of IVT treatment. Similar to our results, GDNF was found to be increased during the acute phase of an IS and remained elevated in the follow-up measurements when compared to controls without clinical atherothrombotic disease [66] or to healthy individuals [67]. However, another study reported a decrease in GDNF of stroke patients compared to controls with cerebrovascular disease [68]. Aside from the astrocytes, GDNF targets other neuronal subpopulations, including central noradrenergic neurons, spinal motoneurons, and various peripheral neurons, such as sympathetic, parasympathetic, sensory, and enteric neurons. Outside the nervous system, it plays a crucial role as a morphogen in kidney development and regulates the differentiation of spermatogonia [69,70]. This may explain why there were no differences between the patients and controls regarding TEV GDNF. Furthermore, when only the TEV population is collected, changes in the initial protein levels may be undetectable, as a small population of cells is often affected in the initial phase of the disease [71].

We also observed a correlation between the NIHSS score at 24 h and ADEV GDNF at this time point and 7 days. In a previously mentioned study, Kurakina et al. reported that the GDNF plasma concentrations were highly predictive of unfavorable functional outcomes and the risk of death during acute periods of an IS [67].

### 3.4. Limitations of the Study

We acknowledge that this study has several limitations that need to be addressed.

Here, we analyzed the cargo of pre-selected subpopulations of EVs derived from astrocytes. Considering this aim, we opted for immunocapture as a method for EV purification. Other techniques, such as transmission electron microscopy and nanoparticle tracking analysis, offer insights into EVs’ size, integrity, and morphology. Instead, immunocapture selectively separates different EV subpopulations in biofluids based on a unique protein on the vesicle surface [72]. The glutamate aspartate transporter (GLAST) is a specific surface marker with the most widespread expression in quiescent and reactive astrocyte subpopulations [73], widely used in astrocyte flow cytometry and sorting [74]. Thereby, we presume that the GLAST-positive EV subpopulation originates from astrocytes, but we cannot exclude the possibility of other sources for these EVs [75]. Immunocapture was followed by Western blot analyses of the EV cargo on a relatively small sample size. This technique was suitable for detecting AQP4 and GDNF, but it proved time-consuming and difficult to implement in a larger patient cohort. Thus, additional methods are required to enhance the accessibility of the determination of EV cargo in these situations. Furthermore, we have not yet established the optimal quality control for the Western blotting of samples containing EVs. In this study, we determined AQP4 and GDNF in the EVs but did not measure their serum/plasma or cerebrospinal fluid levels. We followed our patients for one month, but a longer follow-up would be beneficial, as it might reveal other aspects regarding the initial biomarker levels and recovery patterns.

## 4. Materials and Methods

### 4.1. Patient Enrollment and Study Design

Eighteen patients admitted within 24 h from symptoms onset with acute middle cerebral artery stroke to the 1st and 2nd Neurology Clinics of the County Emergency Clinical Hospital of Targu Mures, Romania, with a clinical stroke severity of 6–11 on the NIHSS score, regardless of whether they received IVT or any previous treatments, were enrolled in this prospective, observational study between December 2021 and May 2023. The exclusion criteria were intracranial hemorrhage; hemorrhagic transformations of IS; stroke-mimic pathologies; transient ischemic attack; history of a previous IS within 12 months; treatment with mechanical thrombectomy; dementia; acute infection; associated serious health conditions, e.g., acute myocardial infarction, liver or renal failure, hematologic or active oncological disease.

AIS diagnosis was established in the emergency department by the on-call neurologist according to the current stroke guidelines. The patients were evaluated at 24 h (D1), 7 days (D7), and one month (M1: day 30  ±  3) following symptoms onset. Clinical stroke severity was estimated using the NIHSS score [76]. Functional outcome was defined according to the mRS [77]. A control CT scan was performed at D1 and D7 to rule out a potential hemorrhagic transformation. The data collected included demographic and clinical information (stroke risk factors, neurological exam, NIHSS score at admission, and during the follow-up period, mRS score at D7 and M1). Nine control subjects with similar demographic characteristics and cardiovascular risk factor profiles to the target group were also recruited. They had no brain lesions and were free from inflammatory or infectious diseases.

The study protocol was approved by the Ethics Committee for Scientific Research of the “George Emil Palade” University of Medicine, Pharmacy, Science and Technology of Targu Mures (renewed approval no. 2303/26.04.2023), which requires all human studies to be conducted in accordance with the Declaration of Helsinki. Before inclusion in the study, written informed consent was obtained from all patients (or their legal representatives/family members).

### 4.2. Isolation and Characterization of EVs and Purification of ADEVs from Plasma Samples

The process of blood collection, followed by the isolation of EVs from plasma samples and the purification of ADEVs using bead-based flow cytometry, were previously presented in detail in our recent study [38].

#### 4.2.1. Isolation of EVs from Plasma Samples

Briefly, peripheral venous blood samples were collected at D1, D7, and M1 after IS onset and centrifuged within 2 h in two stages to obtain the plasma required to isolate the EVs further using the ExoQuick^®^ ULTRA EV precipitation kit (System Biosciences, Palo Alto, CA, USA, cat. no. EQULTRA-20A-1) according to the manufacturer’s protocols [78]. This kit includes purification columns to reduce the IgG and albumin levels in the precipitated and resuspended EV pellets, allowing for a biochemically cleaner examination of these vesicles.

#### 4.2.2. Characterization of EVs Using Bead-Based Flow Cytometry

**Figure 5:**
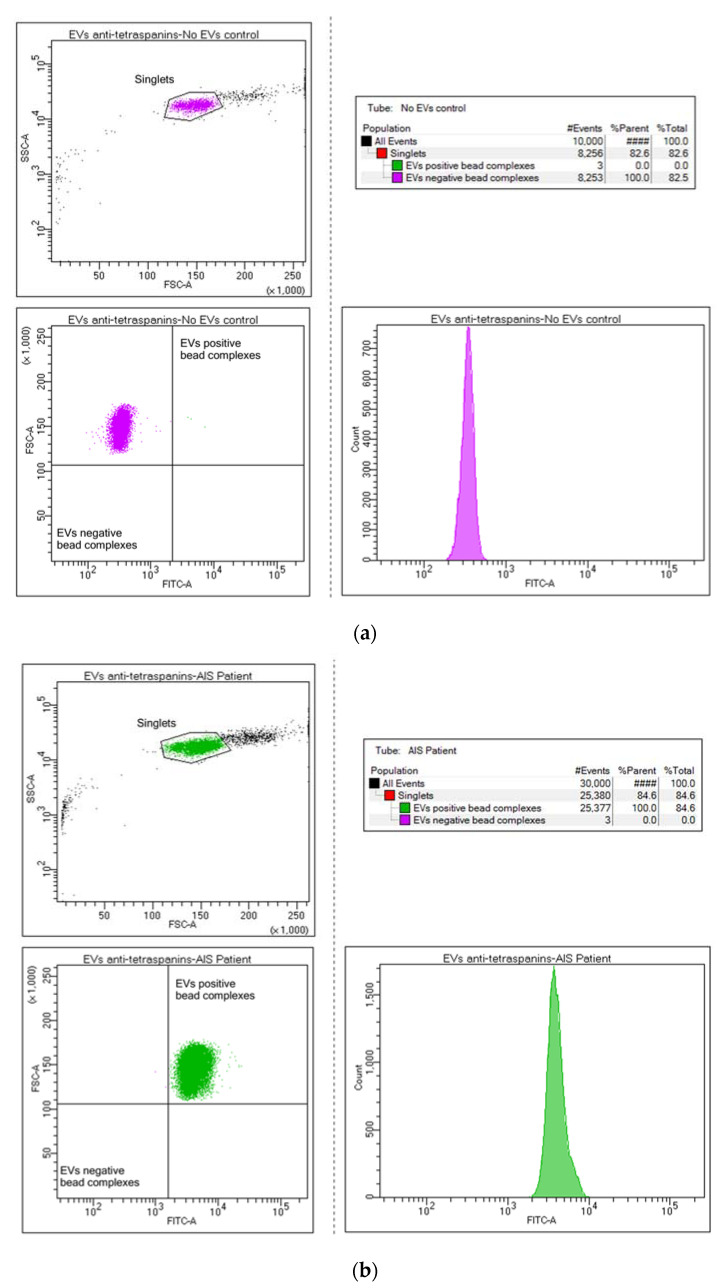
Bead flow separation data for the anti-tetraspanin antibodies coupled with Exo-FITC staining showing beads with no captured EVs (**a**) and beads with captured EVs = tetraspanin-positive (CD9, CD63, and CD81) EVs population (**b**).

To validate the success of the isolation procedure, the obtained EVs were captured using Exo-Flow streptavidin magnetic beads from the Basic Exo-Flow Capture kit (System Biosciences, Palo Alto, CA, USA, cat. no. CSFLOWBASICA-1) coupled with a cocktail of selected biotinylated antibodies targeting CD9 (Miltenyi Biotec, Bergisch Gladbach, Germany, cat. no. 130-103-954), CD63 (Miltenyi Biotec, Bergisch Gladbach, Germany, cat. no. 130-100-169), and CD81 (Miltenyi Biotec, Bergisch Gladbach, Germany, cat. no. 130-122-217), also called tetraspanins, according to the manufacturer’s protocols [79]. To visualize the bead-immobilized EVs, the obtained complexes were stained with Exo-FITC Exosome FACS stain and then analyzed in the BD FACSAria™ III flow cytometer (BD Biosciences, San Jose, CA, USA). The antibody coupled beads without captured EVs were used as negative controls. Flow cytometry data were processed using the BD FACSDiva™ v8.0 Software (BD Biosciences, San Jose, CA, USA) (Figure 5).

#### 4.2.3. Purification of ADEVs Using Bead-Based Flow-Cytometry

**Figure 6:**
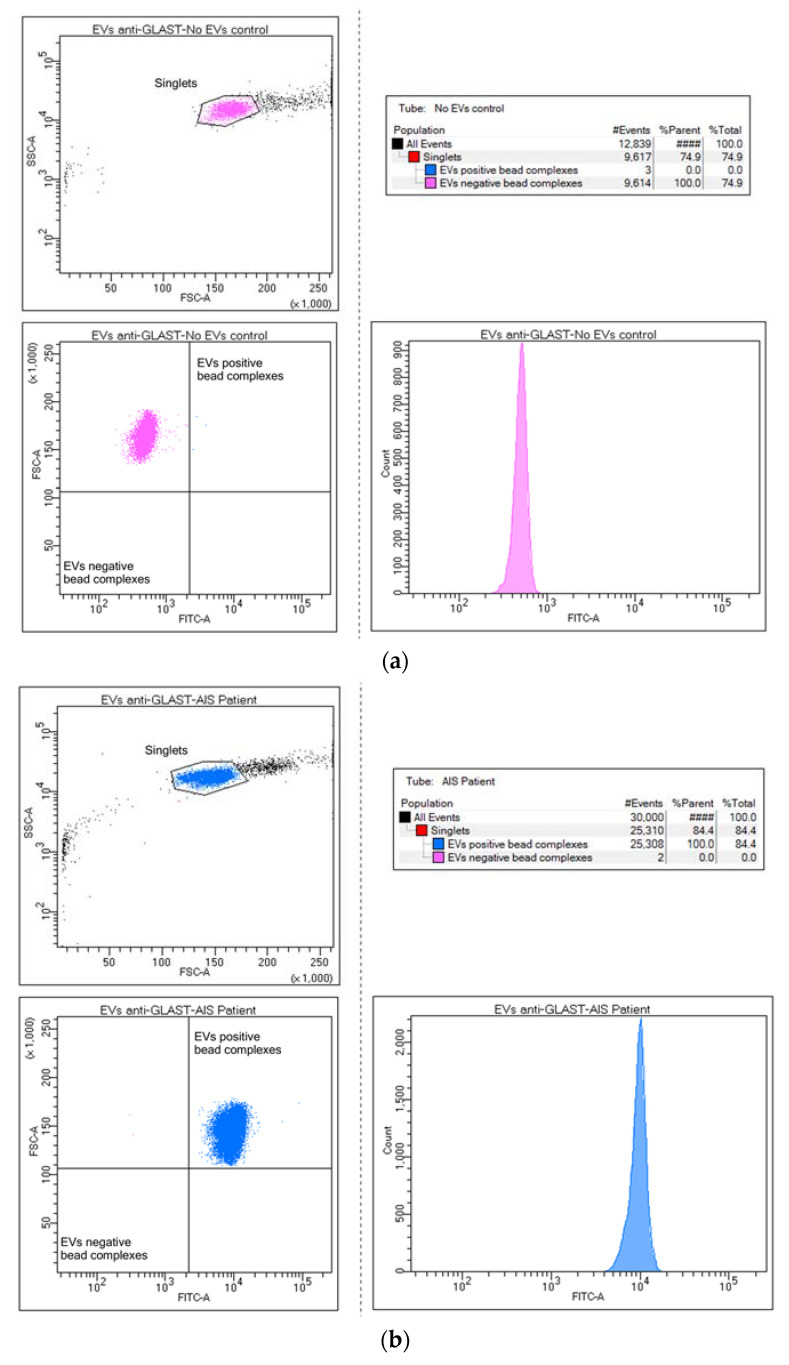
Bead flow separation data for the anti-GLAST antibody coupled with Exo-FITC staining showing beads with no captured EVs (**a**) and beads with captured EVs = GLAST-positive EVs population (**b**).

To isolate the presumed ADEV subpopulation, EVs were captured using anti-GLAST (Miltenyi Biotec, Bergisch Gladbach, Germany, cat. no. 130-118-984) antibody-coupled magnetic beads, following the previously applied protocol (Figure 6).

After the flow sort, we added the Exosome Elution Buffer to simultaneously remove the Exo-FITC stain and elute the intact GLAST-positive EVs from the beads. The resulting purified presumed ADEV suspensions were frozen and stored at −80 °C until further use for downstream analyses.

### 4.3. Characterization of EVs Using Transmission and Scanning Electron Microscopy

**Figure 7:**
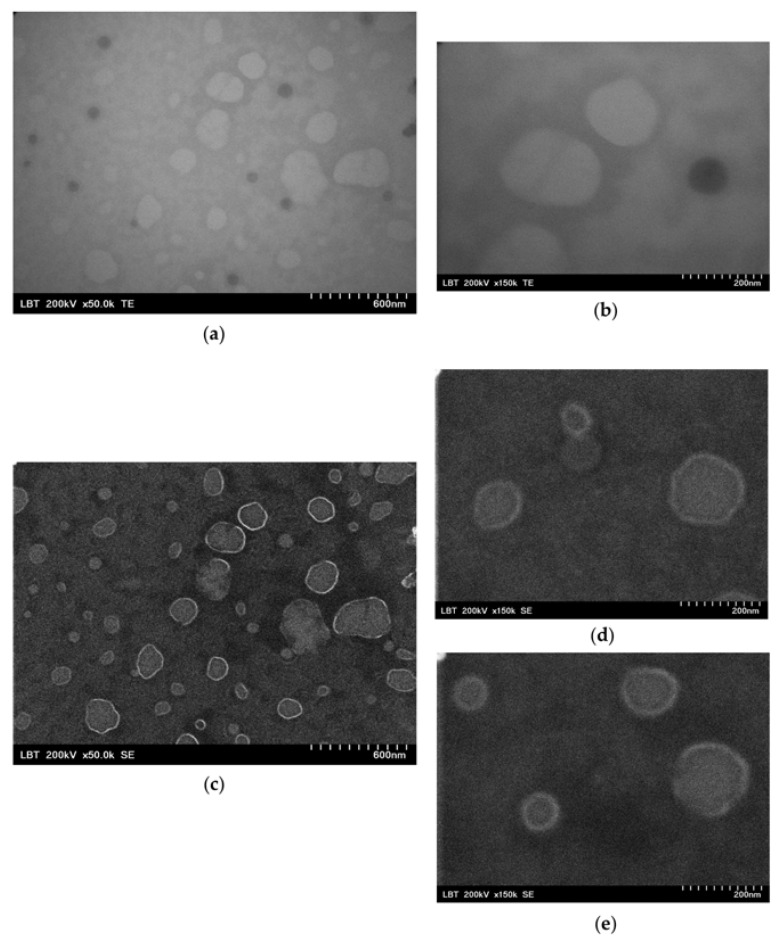
Transmission (TE, (**a**,**b**)) and scanning electron micrographs (SE, (**c**–**e**)) of the isolated EVs shown at two different magnifications of 50,000× (**a**,**c**) and 150,000× (**b**,**d**,**e**). Multiple vesicles with typical EV morphology can be seen in each image.

EVs stored frozen at −80 °C were briefly thawed and mixed with an equal volume of glutaraldehyde to achieve a final concentration of 2.5%. Then, 5 µL of the fixed EVs were placed on Formvar-carbon-coated grids. The EVs were observed and imaged under a Hitachi HD-2700 (Hitachi High-Technologies Corp., Tokyo, Japan) scanning transmission electron microscope without any contrast enhancement at 200 kV, using the magnifications of 50,000× and 150,000×, respectively. The observed nanoscale particles were small and round, typically ranging from 30 to 200 nanometers (nm) for the exosomes and from 100 to 1000 nm for larger EVs like microvesicles. The EVs displayed an outer boundary and presented different contrast levels due to membrane thickness or internal contents, as seen in Figure 7.

### 4.4. Western Blot Quantitative Analyses of EV Proteins

A Western blot assay was conducted to detect and quantify AQP4 and GDNF in EV cargo. After the lysis of EV suspensions with an equal volume of ice-cold protease inhibitor phenylmethylsulfonyl fluoride (Abcam, Cambridge, UK, cat. no. ab141032)-enriched RIPA buffer (Abcam, Cambridge, UK, cat. no. ab156034), the protein amount was measured by a method developed by Iwata and Nishikaze [80]. The mean protein concentration was 167.52 μg/mL (ranging from 17.86 to 508.41 μg/mL) in the TEVs and 21.52 μg/mL (ranging from 3.7 to 62.13 μg/mL) in the GLAST-positive samples. Next, the lysates were mixed with an equal volume of 2× Laemmli Sample Buffer (Bio-Rad Laboratories, Hercules, CA, USA, cat. no. #1610737) and β-mercaptoethanol (Bio-Rad Laboratories, Hercules, CA, USA, cat. no. #1610710) and heated for two minutes at 95 °C.

A total of 50 µL lysate was loaded into wells from 10% Mini-PROTEAN^®^ TGX Stain-Free™ Protein Gels (Bio-Rad Laboratories, Hercules, CA, USA, cat. no. #4568034, 10 wells). Proteins from total and GLAST-positive EV aliquots were separated by SDS-PAGE (sodium dodecyl-sulfate polyacrylamide gel electrophoresis) with Tris/Glycine/SDS running buffer (Bio-Rad Laboratories, Hercules, CA, USA, cat. no. #1610732) in the Mini-PROTEAN^®^ Tetra Vertical Electrophoresis Cell System (Bio-Rad Laboratories, Hercules, CA, USA). Then, the separated proteins were electrophoretically transferred from the gels to polyvinylidene difluoride (PVDF) membranes using the Trans-Blot^®^ Turbo™ Transfer Pack (Bio-Rad Laboratories, Hercules, CA, USA, cat. no. #1704156) with the Trans-Blot^®^ Turbo™ Transfer System (Bio-Rad Laboratories, Hercules, CA, USA). Following a blocking step to diminish non-specific binding, the target EV proteins were detected through overnight incubation using specific primary rabbit monoclonal anti-AQP4 (Thermo Fisher Scientific*,* Waltham, MA, USA, Invitrogen, cat. no. MA5-35079) and anti-GDNF (Thermo Fisher Scientific, Waltham, MA, USA, Invitrogen*,* cat. no. MA5-33142) antibodies, followed by incubation with the peroxidase-conjugated goat anti-rabbit secondary antibody (Goat anti-Rabbit IgG (H + L) Cross-Adsorbed Secondary Antibody, HRP, Thermo Fisher Scientific, Waltham, MA, USA, Invitrogen, cat. no. G-21234). The chemiluminescent detection in the presence of luminol (Clarity™ Western ECL Substrate kit, Bio-Rad Laboratories, Hercules, CA, USA, cat. no. #1705061), image analysis, and densitometric quantification of the band intensities were performed using the ChemiDoc XRS+ System (Bio-Rad Laboratories, Hercules, CA, USA) and ImageLab™ Software version 6.1.0 (Bio-Rad Laboratories, Hercules, CA, USA).

### 4.5. Statistical Analyses

Statistical analyses were performed using GraphPad Prism^®^ Software version 10.3.0 for macOS (GraphPad Software, Boston, MA, USA) and Microsoft^®^ Excel for Mac version 16.83. Data for the descriptive statistics are reported as the median and IQR (Q1–Q3), median and min–max values, mean ± standard deviation, or as the absolute number (n) and percentage (%). Standard differences (d) were used to compare the baseline characteristics of the study population. A d-value of < 0.1 is considered ideal; however, in small biomarker studies (n ≤ 20), an additional or missing variable between the two groups that are compared can lead to a d-value of ≥0.1 [81]. Non-parametric Mann–Whitney U and Friedman’s ANOVA tests (post hoc Dunn’s test) were performed to assess the continuous variables. The Spearman’s rank correlation coefficient (r) was used to determine any relationship between the amount of EV proteins (the AQP4 and GDNF band intensities at D1, D7, and M1) and the stroke severity (NIHSS at D1, D7, and M1), as well as the outcome (mRS at D7 and M1). A *p*-value of < 0.05 was considered significant.

## 5. Conclusions

EVs contain a variety of cargo molecules that may peripherally reflect the brain’s response to different disease-induced changes, unlocking a new form of liquid biopsy. The presence of AQP4 and GDNF in post-ischemic ADEV cargo highlights the importance of EV protein profiling in understanding the astrocytic response to ischemia. These ADEV proteins could potentially serve as biomarkers for stroke progression, particularly at 24 h following symptom onset, providing a promising non-invasive avenue for monitoring IS patients. While these findings are a step forward in stroke research, future studies are needed to elucidate the potential of ADEVs and their cargo proteins as biomarkers for stroke recovery and outcomes and for guiding personalized therapy for an IS.

## Figures and Tables

**Table 1:** Baseline characteristics of the study’s population.

Variables		AIS Patients (n = 18)	Controls (n = 9)	Standardized Difference (d)
Sex	Male	10 (55.6%)	5 (55.6%)	0.00
	Female	8 (44.4%)	4 (44.4%)	
Vascular risk factors	Hypertension	18 (100%)	6 (66.6%)	1
	Hyperlipidemia	4 (22.2%)	2 (22.2%)	0.00
	Diabetes	9 (50%)	4 (44.4%)	0.11
	Atrial fibrillation	3 (16.6%)	1 (11.1%)	0.16
	Current smoker	3 (16.6%)	2 (22.2%)	0.14

Data are n (%). Abbreviation: AIS–acute ischemic stroke.

**Table 2:** Correlations between EV AQP4, EV GDNF, stroke severity, and outcome.

EV Protein		Scales	NIHSS						mRS			
		Time Point	D1		D7		M1		D7		M1	
			r	*p*	r	*p*	r	*p*	r	*p*	r	*p*
AQP4	TEV	D1	0.15	0.529	−0.04	0.869	−0.20	0.515	0.03	0.885	−0.20	0.553
		D7	0.09	0.716	0.24	0.332	−0.04	0.904	0.20	0.407	0.06	0.872
		M1	0.23	0.468	−0.15	0.619	−0.04	0.904	−0.05	0.862	−0.06	0.872
	ADEV	D1	0.50	0.031 *	0.09	0.711	−0.27	0.382	−0.01	0.944	−0.13	0.706
		D7	0.20	0.423	0.22	0.361	0.01	0.958	0.20	0.423	−0.10	0.789
		M1	0.26	0.393	0.05	0.871	0.06	0.850	0.42	0.177	−0.20	0.553
GDNF	TEV	D1	0.37	0.123	0.02	0.908	0.02	0.932	0.29	0.229	0.03	0.961
		D7	0.40	0.096	0.30	0.213	0.04	0.886	0.37	0.125	0.10	0.789
		M1	0.42	0.169	0.20	0.512	0.11	0.727	0.37	0.227	−0.03	0.961
	ADEV	D1	0.49	0.035 *	−0.11	0.651	0.15	0.625	−0.21	0.383	0.10	0.789
		D7	0.53	0.021 *	−0.03	0.889	0.46	0.129	−0.10	0.667	0.34	0.298
		M1	0.52	0.082	0.36	0.238	0.49	0.107	0.34	0.279	0.54	0.079

Spearman correlation, r—coefficient; D1, D7: n = 18; M1: n = 12; * *p*  <  0.05. Abbreviations: NIHSS—National Institutes of Health Stroke Scale; mRS—modified Rankin Scale.

## Data Availability

Data are contained in the article and are available upon request from the corresponding author.

## Abbreviations

ADEVs	astrocyte-derived extracellular vesicles
AIS	acute ischemic stroke
AQP4	aquaporin-4
BBB	blood–brain barrier
C	control
cat. no.	catalog number
CNS	central nervous system
CT	computed tomography
d	standardized difference
e.g.,	for example
EVs	extracellular vesicles
GDNF	glial cell line-derived neurotrophic factor
GFAP	glial fibrillary acidic protein
GLAST	glutamate aspartate transporter
IQR	interquartile range
IS	ischemic stroke
IVT	intravenous thrombolysis
kDa	kilodaltons
mRS	modified Rankin Scale
MW	molecular weight
NIHSS	National Institutes of Health Stroke Scale
Nm	nanometers
Ns	not significant
OAPs	orthogonal arrays of particles
PAGE	polyacrylamide gel electrophoresis
PVDF	polyvinylidene difluoride
RAs	reactive astrocytes
SDS	sodium dodecyl sulfate
SE	scanning electron microscopy
TE	transmission electron microscopy
TEVs	total extracellular vesicles
t-PA	tissue Plasminogen Activator
